# Low Resting Membrane Potential and Low Inward Rectifier Potassium Currents Are Not Inherent Features of hiPSC-Derived Cardiomyocytes

**DOI:** 10.1016/j.stemcr.2018.01.012

**Published:** 2018-02-08

**Authors:** András Horváth, Marc D. Lemoine, Alexandra Löser, Ingra Mannhardt, Frederik Flenner, Ahmet Umur Uzun, Christiane Neuber, Kaja Breckwoldt, Arne Hansen, Evaldas Girdauskas, Hermann Reichenspurner, Stephan Willems, Norbert Jost, Erich Wettwer, Thomas Eschenhagen, Torsten Christ

**Affiliations:** 1 Department of Experimental Pharmacology and Toxicology, University Medical Center Hamburg-Eppendorf, Institut für Experimentelle Pharmakologie und Toxikologie, Universitätsklinikum Hamburg-Eppendorf, Martinistrasse 52, 20246 Hamburg, Germany; 2 DZHK (German Centre for Cardiovascular Research), Partner Site Hamburg/Kiel/Lübeck, 20246 Hamburg, Germany; 3 Department of Cardiology-Electrophysiology, University Heart Center Hamburg, 20246 Hamburg, Germany; 4 Department of Cardiovascular Surgery, University Heart Center Hamburg, 20246 Hamburg, Germany; 5 Department of Pharmacology and Pharmacotherapy, Faculty of Medicine, University of Szeged, 6721 Szeged, Hungary; 6 Institute of Pharmacology, University Duisburg-Essen, 45122 Essen, Germany

**Keywords:** human induced pluripotent stem cell-derived cardiomyocytes, inward rectifier K^+^ current, I_K1_, I_K,ACh_, engineered heart tissue, resting membrane potential, repolarization fraction, action potential duration, human atrium, human ventricle

## Abstract

Human induced pluripotent stem cell (hiPSC) cardiomyocytes (CMs) show less negative resting membrane potential (RMP), which is attributed to small inward rectifier currents (I_K1_). Here, I_K1_ was measured in hiPSC-CMs (proprietary and commercial cell line) cultured as monolayer (ML) or 3D engineered heart tissue (EHT) and, for direct comparison, in CMs from human right atrial (RA) and left ventricular (LV) tissue. RMP was measured in isolated cells and intact tissues. I_K1_ density in ML- and EHT-CMs from the proprietary line was similar to LV and RA, respectively. I_K1_ density in EHT-CMs from the commercial line was 2-fold smaller than in the proprietary line. RMP in EHT of both lines was similar to RA and LV. Repolarization fraction and I_K,ACh_ response discriminated best between RA and LV and indicated predominantly ventricular phenotype in hiPSC-CMs/EHT. The data indicate that I_K1_ is not necessarily low in hiPSC-CMs, and technical issues may underlie low RMP in hiPSC-CMs.

## Highlights

• Here, we report I_K1_ current density in hiPSC-CMs as high as in human adult CMs
• Physiological RMP was found with sharp microelectrodes in engineered heart tissue
• RMP measured by the patch-clamp technique in hiPSC-CMs may be underestimated
• RMP and APD_90_ are not sufficient to categorize CMs as atrial or ventricular

## Teaser

We show here that RMP is systematically underestimated in patch-clamped hiPSC-CMs and, in the 3D EHT format, reaches physiological values of human adult CMs when measured by sharp microelectrodes. This corresponds with I_K1_ currents as large as in human adult CMs. In human adult preparations, repolarization fraction was more useful than APD and RMP to classify action potentials as atrial or ventricular like.

## Introduction

Human induced pluripotent stem cell-derived cardiomyocytes (hiPSC-CMs) are promising tools for cardiac repair (Weinberger et al., 2016), disease modeling (Burridge et al., 2011, Itzhaki et al., 2011, Liang et al., 2013, Liang et al., 2016, Moretti et al., 2010, Yazawa et al., 2011, Zhang et al., 2014), and cardiovascular drug testing (Eder et al., 2014, Kijlstra et al., 2015, Liang et al., 2013, Lu et al., 2015, Ma et al., 2011, Maddah et al., 2015). However, there is concern that hiPSC-CMs differ in their electrophysiological properties from human adult CMs. Studies with patch-clamp electrodes consistently reported low resting membrane potentials (RMPs) in hiPSC-CMs compared with adult atrial or ventricular myocardium (Chen et al., 2017, Davis et al., 2012, Doss et al., 2012, Herron et al., 2016, Karakikes et al., 2015, Liang et al., 2013, Liang et al., 2016, Ma et al., 2011, Vaidyanathan et al., 2016). This is an alarming finding since correct RMP is mandatory for excitability and refractoriness. One of the possible explanations for a slightly negative RMP reported in hiPSC-CMs is related to the inward rectifier K^+^ current (I_K1_). This current maintains the stable RMP in adult CMs (Hibino et al., 2010). In line with this assumption, current density of I_K1_ were reported to be low, or almost absent, in hiPSC-CM cell lines (Doss et al., 2012, Herron et al., 2016, Ma et al., 2011). Consequently, sophisticated electronic approaches based on dynamic patch clamping or overexpression of channel subunits of I_K1_ were proposed to compensate low current density in hiPSC-CMs (Meijer van Putten et al., 2015, Vaidyanathan et al., 2016). On the other hand, capacitance of hiPSC-CMs is smaller compared with adult CMs, and patch-clamp-based determination of the membrane potential in small cells has been associated with methodological problems (Amos et al., 1996, Wilson et al., 2011). These findings raise the hypothesis that part of the reported differences between hiPSC-CM and adult human heart electrophysiology are in fact methodically in nature.

## Results

### hiPSC-CMs Show Robust Inward Rectifier Potassium Currents

**Figure 1:**
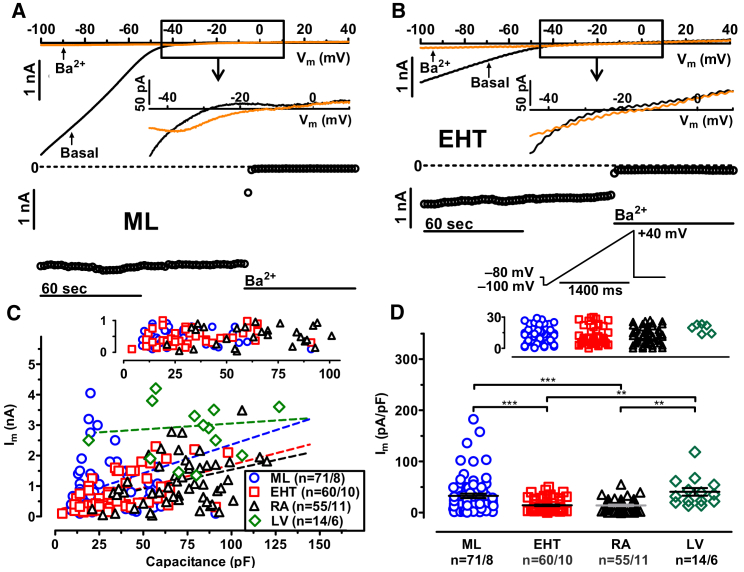
Cell Size and Inward Rectifier Potassium Current in hiPSC-CMs from ML, EHT, RA, and LV (A and B) Original traces of inward rectifier currents (I_K1_) and time courses of current at −100 mV in hiPSC-CMs obtained from ML and EHT exposed to 1 mM Ba^2+^. Outward component of I_K1_ and voltage protocol is given as inset in (B). (C) Ba^2+^-sensitive I_K1_ current amplitudes measured at −100 mV plotted against cell capacitance; dotted lines indicate linear regression fit (R^2^ values were 0.1 for ML, 0.28 for EHT, 0.04 for iCell-EHT, 0.15 for RA, and 0.007 for LV). The upper scale shows data points with lower current amplitude. (D) Individual data points and respective mean values ± SEM for Ba^2+^-sensitive I_K1_ current densities measured at −100 mV in hiPSC-CMs from ML, EHT, and in CMs from RA and LV. The upper scale shows data points with lower current density. ^∗∗^p < 0.01, ^∗∗∗^p < 0.001 (one-way ANOVA followed by Bonferroni test). n/n, number of experiments/number of isolations in hiPSC-CMs and number of experiments/number of patients in RA and LV.

To detect inward rectifier currents, a classical ramp protocol was applied (Figure 1B, inset). High extracellular potassium concentration (20 mM) was used to evoke large inward currents even at moderately negative test pulse potentials, but also to facilitate larger outward currents (Figures 1A and 1B) (Anumonwo and Lopatin, 2010). It is also reported that applying higher external K^+^ concentration can reduce leakage and improve the stability of the measurements (Wilson et al., 2011). We found clear evidence for inwardly rectifying currents not only in every adult CM (left ventricular [LV] and right atrial [RA]), but also in every hiPSC-CM from both monlayer (ML) and engineered heart tissue (EHT) (Figure 1C). There was a large variability in current size, with some currents close to zero, not only in ML and EHT, but also in RA (Figure 1C). However, we found in every cell from RA, LV, ML, and EHT an instantaneous block of inward current in response to Ba^2+^ (1 mM). To investigate whether larger cells show a more mature phenotype with larger I_K1_, we plotted individual inward current amplitudes versus cell size (Figure 1C). As shown before (Liang et al., 2013, Uzun et al., 2016), hiPSC-CMs, isolated from ML or EHT, were smaller than RA and LV CMs (30.8 ± 1.5 pF, n = 167 in ML, 47.0 ± 1.7 pF, n = 260 in EHT, 73.6 ± 4.0 pF, n = 55 in RA, 88.8 ± 13.2 pF, n = 15 in LV). However, there was only a weak positive association between current amplitude and cell membrane capacitance. The steepness of the regression lines were low in all groups, and did not differ between hiPSC-CMs and human adult CMs (Figure 1C, p = 0.6, one-way ANOVA). Statistical significance for slope different from zero was only determined in ML, EHT, and RA (R^2^ values were 0.09 for ML, 0.28 for EHT, and 0.14 for RA). This argues against the assumption that smaller hiPSC-CMs represent a more immature phenotype with smaller I_K1_ current amplitude. To facilitate comparison with other publications, we present current amplitudes normalized to cell size. Current densities in ML were not significantly smaller than in LV (Figure 1D; Table S1). Current density of I_K1_ in EHT was smaller than in ML and LV, but still reached the values of RA (Figure 1D; Table S1). Thus, I_K1_ current densities in hiPSC-CMs were not lower than in human adult CMs, when identical patch-clamp protocols were applied.

### I_K1_ in hiPSC-CMs Is Conducted by Highly Ba^2+^-Sensitive Kir Channels

**Figure 2:**
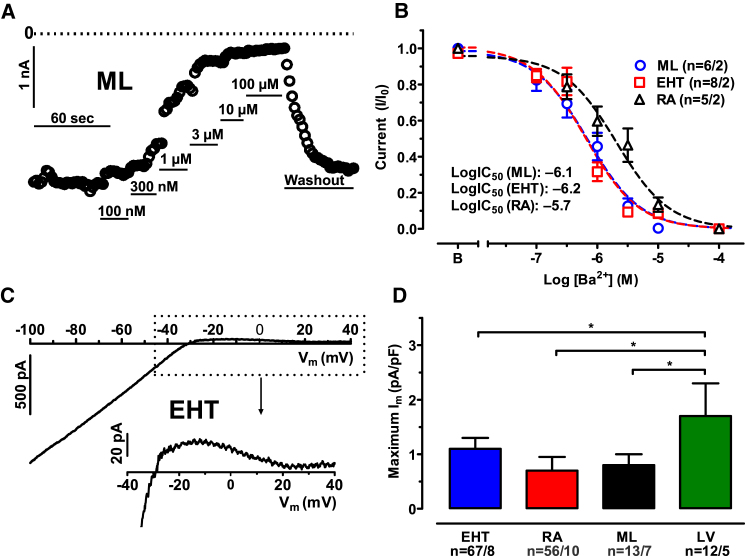
Ba^2+^ Sensitivity and Outward Component of I_K1_ in hiPSC-CMs and Human Adult CMs (A) Time course of inward currents measured at −100 mV in a hiPSC-CMs from ML exposed to increasing concentrations of Ba^2+^. (B) Concentration-response curves for Ba^2+^ on inward current at −100 mV in hiPSC-CMs from ML, EHT, and in CMs from RA. Mean values ± SEM. (C) Ba^2+^-sensitive current trace from hiPSC-CMs from ML. Outward component of current given on an extended scale at the bottom. (D) Mean values ± SEM of maximum outward peak currents in hiPSC-CMs from ML, EHT, and in CMs from RA and LV. ^∗^p < 0.05 (one-way ANOVA followed by Bonferroni test) n/n = number of experiment/number of isolations in hiPSC-CMs and number of experiment/number of patients in RA and LV.

The I_K1_ conducting channel exists as tetramer, assembled from different α subunits (Kir2.1–2.4; for review see Hibino et al., 2010). Different channel-forming subunits of I_K1_ show different sensitivity to Ba^2+^ (Liu et al., 2001, Schram et al., 2003). Heart muscle expresses Kir2.1, 2.2, and 2.3, which show high sensitivity to Ba^2+^ (in the low μM range) (Schram et al., 2003). Kir2.4 exhibits lower Ba^2+^ sensitivity and is expressed in neuronal tissue only (Liu et al., 2001). To elucidate whether I_K1_ in hiPSC-CMs is conducted by the cardiac subunits, we measured concentration-response curves for Ba^2+^ block on the inward I_K1_ current (Figure 2A). We observed a monophasic concentration-response curve, arguing against the contribution of Kir2.4 (Figure 2B). I_K1_ in hiPSC-CMs from both culture conditions showed higher Ba^2+^ sensitivity than in RA: the logIC_50_ values for Ba^2+^ were −6.09 (95% confidence interval [CI]: −6.27 to −5.91) in ML, versus −6.15 (CI: −6.32 to −5.99) in EHT, versus −5.66 in RA (CI: –5.92 to −5.41, p < 0.01, F test for ML versus RA and for EHT versus RA; Figure 2B). The qRT-PCR data confirmed that the cardiac isoforms of the Kir channels (2.1–2.3) are expressed in hiPSC-CMs (Figures S1A–S1C), while Kir2.4 is not (Figure S1D).

### I_K1_ in hiPSC-CMs Shows Small Outward Contribution

In cardiac myocytes, the outward component of I_K1_ contributes to the late phase of the repolarization. There is no simple relationship between the size of inward and outward currents: the relative outward contribution of I_K1_ is larger in ventricular than in atrial CMs from human hearts (Amos et al., 1996, Koumi et al., 1995, Varró et al., 1993, Wang et al., 1998). The outward component of I_K1_ is generally larger in rabbit and canine than in human(Amos et al., 1996, Jost et al., 2013, Koumi et al., 1995, Major et al., 2016, Varró et al., 1993, Wang et al., 1998). Overall, contribution of I_K1_ to overall repolarization in human heart is small and restricted to the late phase of action potential (AP) (Jost et al., 2013). Therefore, we measured the maximum outward current density of I_K1_ during ramp pulses (Figure 2C). As shown before, in human CMs, outward currents conducted via I_K1_ were small compared with inward currents and smaller in human atrial than in ventricular CMs (0.7 ± 0.25 pA/pF versus 1.7 ± 0.6 pA/pF, n = 16 and 13; Figure 2D). Outward currents in hiPSC-CMs were as small as in RA (1.1 ± 0.2 pA/pF, n = 67 in ML, 0.8 ± 0.2 pA/pF, n = 56 in EHT; Figure 2D). Thus, hiPSC-CMs from both ML and EHT showed small outward contribution of I_K1_ as known for human adult CMs. The small contribution of I_K1_ to repolarization may favor stronger action potential duration (APD) prolongation upon block of I_Kr_ (Jost et al., 2013). Therefore, prediction of QT prolongation in human by hERG blockers could be more meaningful, when hiPSC-CMs are used instead of canine or rabbit. Further studies are needed to investigate whether hiPSC-CMs quantitatively reflect the repolarization reserve in human heart.

### hiPSC-CMs Do Not Express Acetylcholine-Activated Potassium Currents

**Figure 3:**
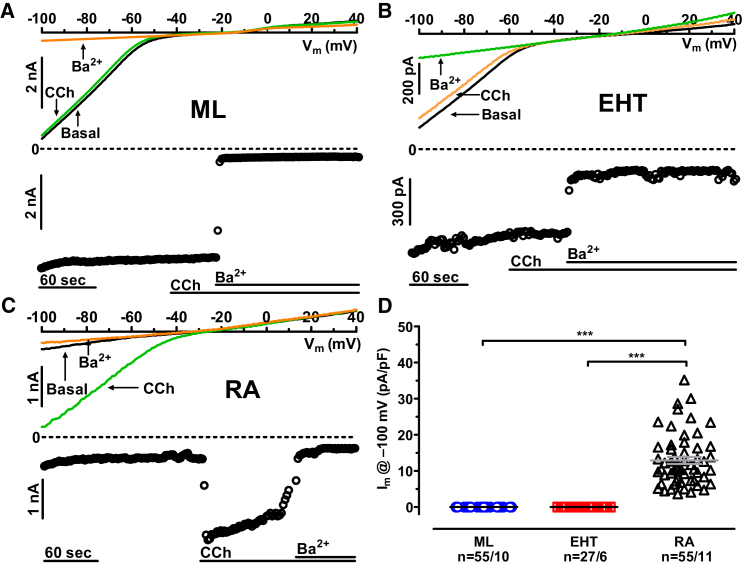
Lack of Carbachol Effect on Inward Rectifiers in hiPSC-CMs (A and B) Original traces of inward rectifier currents and time courses of current at −100 mV in hiPSC-CMs obtained from ML and EHT exposed to carbachol ([CCh], 2 μM). (C) Effect of CCh (2 μM) on I_K,ACh_ in CMs from RA. (D) CCh effects expressed as absolute current change in response to CCh (2 μM) in ML, EHT, and in CMs from RA. Individual data points and mean values ± SEM. LV ^∗∗∗^p < 0.001 (one-way ANOVA followed by Bonferroni test). n/n = number of experiment/number of isolations in hiPSC-CMs and number of experiment/number of patients in RA.

The existence of acetylcholine-activated potassium currents (I_K,ACh_) is a hallmark of atrial tissue (Dobrzynski et al., 2001). To assess the specification of hiPSC-CMs, we applied the muscarinic (M2) receptor agonist carbachol ([CCh], 2 μM; Figure 3A). In RA CMs, CCh activated a large inward current (Figure 3C), which was absent in all hiPSC-CMs (ML and EHT; Figures 3A and 3C). On the transcript level, we found large expression of Kir3.1 in RA, but not in LV, ML, or EHT (Figure S1). Interestingly, Kir3.4 was expressed in hiPSC-CMs under both culture conditions at least as high as in RA (Figure S1). The latter finding implies that expression of Kir3.4 in hiPSC-CMs alone is not sufficient to generate I_K,ACh_ (Krapivinsky et al., 1995). The lack of I_K,ACh_ indicates that hiPSC-CMs did not exhibit an atrial phenotype.

### RMP and AP Measurements in Single Cells and Intact Tissues

**Figure 4:**
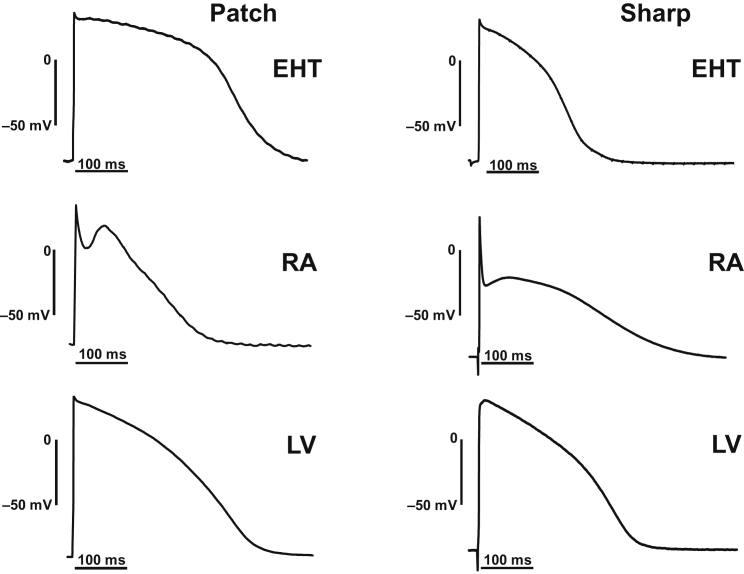
Original AP Recordings Taken from EHT, RA, and LV Action potential recorded by patch-clamp (left) and sharp microelectrode (right) techniques. The patch-clamp recordings were measured from isolated CMs, while the sharp microelectrode recordings were measured from intact muscle preparations.

To decide whether the relatively normal I_K1_ densities observed in hiPSC-CMs result in physiological RMP, we measured action potential in isolated CMs by patch-clamp technique (Figure 4, left column). RMP measured by patch electrodes in hiPSC-CMs was low (−39.3 ± 6.1 mV, n = 12). Application of holding currents was necessary to elicit stable AP in most of the hiPSC-CMs (40 out of 41 in EHT). The amount of holding current was in the range of 0.2 nA. AP could be elicited from a relatively low RMP in EHT (−59.7 ± 1.2 mV, n = 41). In contrast, AP could be recorded in human adult CMs from RA and LV without any holding current, and, in both, RMP was significantly more negative than in EHT. Respective RMP amounted to −74.4 ± 0.5 mV in RA (n = 49) and to −75.9 ± 1.1 mV in LV (n = 10, Figure S2B, upper panel; Table S3).

The sharp microelectrode technique represents the gold standard to measure AP in multicellular preparations. To determine whether the apparent discrepancy between I_K1_ density and RMP in hiPSC-CMs could be related to methodological issues, we compared data from EHT with a larger number of recordings measured in intact RA and LV preparations (Figures 4, right column, 5C, and 5D). The majority of data was collected over many years at the Department of Pharmacology and Toxicology at the Medical Faculty of Dresden University of Technology. All EHTs were beating spontaneously, slightly slower than 60 bpm, which allowed us to record stimulated AP at 1 Hz. RMP in sharp microelectrode recordings was slightly, but significantly less negative in RA than in LV (−73.3 ± 0.3 mV, n = 220 versus −75.9 ± 0.7 mV, n = 57; p < 0.001, Figure S2B, lower panel; Table S3). RMP in intact EHT was in between (−74.6 ± 1.2 mV, n = 24) not significantly different from RA and LV (Figure S2B, lower panel; Table S3). At this point, it should be emphasized that I_K1_ density alone does not determine RMP but the relation of all conductances present near RMP. Two other parameters, action potential duration at 90% repolarization (APD_90_) and repolarization fraction – calculated as (APD_90_−APD_50_)/APD_90_ – were recently proposed as a possible approach to distinguish between atrial- and ventricular-like hiPSC-CMs (Du et al., 2015). It should be noted that impact of I_K1_ on APD_90_ in human LV is very small and repolarization fraction dominated by transient potassium outward currents. Therefore, both parameters are probably rather independent of I_K1_ (Jost et al., 2013). Nevertheless, we aimed to determine if adult human cardiac tissue from RA and LV can be classified correctly and, more importantly, whether hiPSC-CMs represent atrial- or ventricular-like phenotype or a mixture of both.

**Figure 5:**
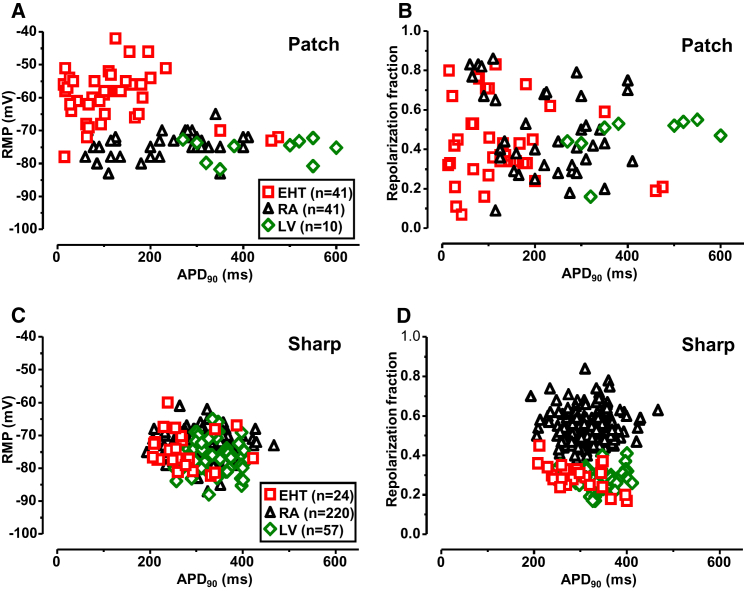
Individual Distribution of APD_90_, RMP, and Repolarization Fraction in EHT, RA, and LV (A and C) Individual data points of APD_90_ plotted versus respective RMP values measured by patch clamping of isolated CMs from EHT, RA, and LV (A) and by sharp microelectrodes in intact tissues from EHT, RA, and LV (C). (B and D) Individual data points of repolarization fraction versus APD_90_ in isolated CMs: from EHT, RA, and LV (B); and in intact tissues: EHT, RA, and LV (D).

When measured by patch electrodes, APD_90_ was shorter in RA than in LV (220 ± 16 ms, n = 41 versus 434 ± 39 ms, n = 10; p < 0.001) and even shorter in hiPSC-CMs (119 ± 17 ms, n = 41; p < 0.001 versus RA p < 0.01 versus LV; Figure S2A, upper panel; Table S2). However, in these two parameters, there was substantial overlap of individual data points between RA and LV (Figures 5A and 5B).

Using sharp microelectrodes, APD_90_ scatter was much lower than in patch-clamp recordings (Figures S2A and 5C). The mean APD_90_ was shorter in RA than in LV (317 ± 3 ms, n = 220 versus 334 ± 6 ms, n = 57; p < 0.05; Figure S2A, lower panel; Table S2). Again, APD_90_ was clearly shorter in EHT (271 ± 11.4 ms, n = 24; p < 0.001 versus RA and p < 0.001 versus LV; Figure S2A, lower panel; Table S2). Next we retrospectively evaluated whether differences in repolarization fraction would be a useful parameter to differentiate between LV and RA. As shown in Figure S2C (bottom panel), a narrow distribution of repolarization fraction was found in AP measurements from intact muscle preparations. There was almost no overlap between LV and RA, indicating the usefulness of the approach (Figure 5D). Repolarization fraction of intact EHTs was similar to LV and differed significantly from RA (EHT: 0.32 ± 0.01, n = 24 versus RA: 0.54 ± 0.01, n = 220 versus LV: 0.28 ± 0.01, n = 57; Figure S2C, bottom panel; Table S4). In contrast to sharp microelectrode recordings, measurements of repolarization fraction in individual CMs showed a wide range of distribution (Figures S2C, upper panel, and 5B).

### Robust RMP in Our hiPSC-CMs: A Peculiarity of a Single-Cell Line?

**Figure 6:**
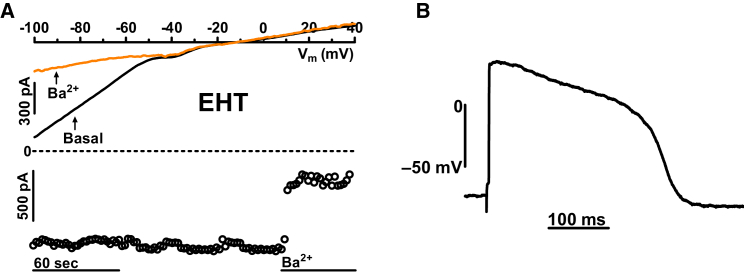
Inward Rectifier Potassium Current and Action Potential in hiPSC-CMs from iCell-EHT (A) Original traces of inward rectifier currents (I_K1_) and time courses of current at −100 mV in hiPSC-CMs obtained from iCell-EHT exposed to 1 mM Ba^2+^. (B) Action potential recorded from iCell-EHT by sharp microelectrode technique. The recordings were measured from intact EHT preparations at 37°C.

We were concerned that normal RMP may be a peculiarity of our proprietary hiPSC-CMs. Therefore we measured I_K1_ densities and AP characteristics in a commercially available cell line (iCell, Cellular Dynamics International, Madison, WI, USA; Figures 6A and 6B). Capacitance of cells was not different from EHT cast from C25 (41.4 pF ± 3.7 pF, n = 32 in iCell-EHT). However, inward I_K1_ density was significantly smaller (7.8 ± 1.8 pA/pF, n = 32; p < 0.05). Nevertheless, by applying sharp microelectrodes, we found a physiological RMP similar to EHT from C25 line (−74.3 ± 0.1 mV, n = 8 for iCell-EHT versus −74.6 ± 1.2 mV, n = 24 for C25-EHT). The data suggest that I_K1_ densities may differ in CMs obtained from different hiPSC lines, but this does not necessarily result in a less negative RMP. Repolarization fraction of iCell-EHT was even lower than LV (0.2 ± 0.01, n = 8 in iCell-EHT versus 0.28 ± 0.01, n = 57; p < 0.05 in LV).

## Discussion

In this study, we directly compare I_K1_ density, RMP, and AP properties in hiPSC-CMs and human atrial and ventricular CMs/tissues under identical experimental conditions. The main findings are (1) that inward I_K1_ current densities and RMP were similar in hiPSC-CMs and human CMs/tissues, (2) that ∼2-fold differences in inward I_K1_ density between CMs from a proprietary and commercial hiPSC line did not translate in significant differences of RMP, and (3) that sharp microelectrode measurements (of intact 3D heart muscle preparations) may provide more reliable data on RMP and repolarization fraction than patch-clamp recordings (of isolated cells). As a side result, in sharp microelectrode recordings repolarization fraction differentiated much better between atrial and ventricular tissue than RMP or APD.

### I_K1_ in hiPSC-CMs and in Adult Human Atrial and Ventricular CMs

I_K1_ density is known to be larger in ventricular than in atrial CMs (Amos et al., 1996, Koumi et al., 1995, Varró et al., 1993, Wang et al., 1998) and very low or absent in nodal cells (Guo et al., 1997, Schram et al., 2002, Tamargo et al., 2004). We found the inward component of I_K1_ to be 2.5-fold larger in LV than in RA CMs. The difference is smaller than reported in some studies (Koumi et al., 1995, Wang et al., 1998), but well in line not only with the report (Varró et al., 1993; small sample size), but also with the largest study published to date (Amos et al., 1996). The observation that our proprietary hiPSC-CMs showed similar inward I_K1_ densities as human CMs was unexpected, given that earlier publications reported low I_K1_ in hiPSC-CMs (Doss et al., 2012, Herron et al., 2016, Ma et al., 2011). Yet, several data argue that true cardiac I_K1_ current was measured. (1) Our data in native human CMs closely reflect reference values as stated above. (2) High Ba^2+^ sensitivity and normal transcript levels of the cardiac ion channel subunits indicate normal I_K1_. (3) The small outward contribution and the absence of a CCh response in hiPSC-CMs (as well as in LV CMs) argues against contribution of I_K,ACh_, an atrial-specific potassium current. (4) We could reproduce relatively low I_K1_ values in the commercially available iCells measured previously (Ma et al., 2011). Thus, we are confident that the data reflect cardiac I_K1_ in hiPSC-CMs.

I_K1_ density in hiPSC-CMs from ML was as high as in human LV CMs. In hiPSC-CMs isolated from EHT, the current density only reached the lower values of RA. As culture of hiPSC-CMs in EHT leads to signs of advanced maturation, this was an unexpected finding, while it was shown before that culturing hiPSC-CMs on different platforms (for example, polydimethylsiloxane) can increase inward I_K1_ density (Herron et al., 2016, Lemoine et al., 2017, Mannhardt et al., 2016). The reasons are not clear at present.

### RMP in hiPSC-CMs and in Adult Human Atrial and Ventricular CMs

**Figure 7:**
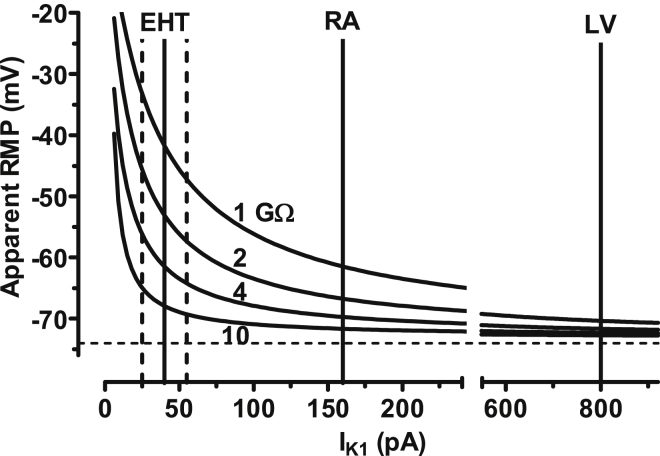
Influence of Seal Resistance and Absolute I_K1_ Amplitude on Apparent RMP in Patch-Clamp Experiments Curves give calculated apparent RMP as a function of absolute I_K1_ amplitudes and seal resistance. It is assumed that inward current amplitudes are determined by I_K1_ only. True RMP was assumed to be −73 mV, as indicated by the dotted horizontal line. Vertical lines indicate absolute I_K1_ amplitudes at −100 mV. Dotted vertical lines illustrate 95% CI of I_K1_ measured in EHT. The apparent RMP was calculated by the equation V_cM_ = V_mM_ + V_mM_^∗^ R_M_/R_seal_. Please note data for EHT are taken from experiments independent from those presented in Figure 1 (numbers given in the Discussion). Data for RA and LV are taken from the literature (Amos et al., 1996). Numbers near the fit curves indicates seal resistance used for calculation. For details, see Discussion.

In line with previous publications we found less negative RMP in isolated hiPSC-CMs, measured by patch-clamp electrodes (Chen et al., 2017, Davis et al., 2012, Doss et al., 2012, Herron et al., 2016, Karakikes et al., 2015, Liang et al., 2013, Ma et al., 2011, Vaidyanathan et al., 2016). In contrast, RMP reached the physiological range when measured in intact EHT by sharp microelectrodes. This discrepancy raised the question whether RMP measurements with patch-clamp electrodes would give a systematic error. The reliability of patch-clamp recordings critically depends on seal resistance (in the range of 1–10 GΩ). The remaining leak current is expected to reduce the actual membrane voltage (Amos et al., 1996, Schneider and Chandler, 1976). The corrected membrane potential (V_cM_) can be calculated from the actual seal resistance (R_seal_), the membrane resistance (R_M_), and the membrane potential, measured during the experiment (V_mM_):

V_cM_ = V_mM_ + V_mM_^∗^ R_M_/R_seal_,

If R_seal_ is considered in series with R_M_. R_seal_ in our experiments was between 1 and 10 GΩ, both in adult CMs and in hiPSC-CMs. R_M_ at the RMP is determined by conductivity via I_K1_. To get an estimate of R_M_ generated by I_K1_ under the same experimental conditions used for AP recordings, we measured the barium-sensitive I_K1_ (1 mM) at physiological [K^+^]_ext_ (5.4 instead of 20 mM) and at physiological temperature (37°C) in hiPSC-CMs from EHT. As expected, current density of I_K1_ was smaller at lower than at higher external K^+^ concentration (1.5 ± 0.7 pA/pF, n = 12 at 5.4 mM K^+^_[ext]_ versus 14.1 ± 12.0 pA/pF, n = 60 at 20 mM K^+^_[ext]_; p < 0.05). Reversal potential shifted to about −70 mV under those conditions. Dividing the actual driving force for potassium (∼30 mV) by the Ba^2+^-sensitive absolute current amplitude measured at −100 mV (41.1 ± 13.7 pA, n = 12) gives a membrane resistance of around 0.75 GΩ. If we assume a true RMP around −73 mV and a typical seal resistance of 3 GΩ, membrane voltage recorded by patch-clamp electrodes will therefore be reduced to a value of −58 mV (according to the equation above). We do not have data for I_K1_ in LV CMs at 5.4 mM K^+^ and at 37°C. Therefore we refer to published data. Mean I_K1_ current densities were 1.1 and 2.8 pA/pF in RA and LV, respectively. Multiplying the values with the capacitances given in that study (145 and 285 pF) we estimated absolute amplitudes of ∼160 pA in atrial and ∼800 pA in ventricular CMs under these conditions (Amos et al., 1996). Plotting V_cM_ versus I_K1_ amplitudes reveals that, even at the same seal resistances in hiPSC-CMs and human adult CMs, errors in hiPSC-CMs will be larger because of their small cell size. Even the 2.5 times higher I_K1_ density in ML may not be sufficient to leave the error zone, since absolute current amplitudes calculates to only 79 pA. For methodological reasons, we cannot provide seal resistance values after getting access to the cells. We found hiPSC-CMs rather fragile, and are afraid that seal resistance could drop down during an experiment. Importantly, in cells with low I_K1_ conductivity, due to a combination of low current density and small cell size, even small changes in seal resistance can have drastic effects on apparent RMP. In addition, our calculations are based on mean values for cell size and I_K1_. Due to large variability both in cell size and I_K1_ density (Figure 1C), underestimation of RMP may be much larger in an individual cell (Figure 7). Therefore, given the present limitations, measurements of AP with patch-clamp pipettes are prone to error and not well suited as an indirect parameter of I_K1_ density. Small cell size is not necessarily an issue, since AP measurements are feasible even in much smaller cells, such as pancreatic cells (Rizzetto et al., 2015), whose size are in the range of 5 pF (Rorsman Patrik, 1986). Very high seal resistance up to 10 GΩ and very large potassium conductance may facilitate AP recording in those cells (Keizer and Magnus, 1989). Sharp microelectrodes can be used to measure AP even in isolated cardiac myocytes (Szentandrássy et al., 2015) as well as in clusters of hiPSC-CMs (Christoforou et al., 2013, Cordeiro et al., 2015, Doss et al., 2012, Zhang et al., 2009). We applied this technique in single hiPSC-CMs, but were able to measure AP in only one single cell from 85 trials (Figure S3). In most cases we had problems to impale the very flat hiPSC-CMs without touching the bottom of the recording chamber.

### Discrimination of Atrial and Ventricular Cells by I_K,ACh_, I_K1_, RMP, APD_90_, and Repolarization Fraction

Earlier reports suggested standard hiPSC-CM cultures contain a mixture of ventricular-, atrial-, and nodal-like CMs (Itzhaki et al., 2011, Liang et al., 2013, Liang et al., 2016, Ma et al., 2011, Ma et al., 2013, Moretti et al., 2010). Classification in these studies was based on differences of AP parameters. Here we have used a different approach based on the presence or absence of I_K,ACh_. Only atrial and nodal CMs express I_K,ACh_ (Heidbüchel et al., 1987, Sakmann et al., 1983, Yamada et al., 1998). We could not find a single hiPSC-CM responding to CCh with a change of inwardly rectifying current. The absence of I_K,ACh_ cannot be explained by a lack of muscarinic receptors, since CCh reverses positive inotropic effects of isoprenaline in the same hiPSC-EHTs (Mannhardt et al., 2016), the classical accentuated antagonism (Levy, 1971). Thus, the data argue against an atrial phenotype of the hiPSC-CMs studied herein.

As outlined above, different I_K1_ densities might also discriminate between atrial and ventricular CMs. However, we found mean I_K1_ densities to differ only moderately between human RA and LV (2.5-fold) and, importantly, to scatter largely and substantially overlap between the groups (Figure 1D). Thus, our data do not support recent suggestions that I_K1_ density should be used to discriminate between atrial and ventricular phenotype in hiPSC-CMs (Giles and Noble, 2016).

A third parameter differing between atrial and ventricular CMs is RMP. In line with the higher I_K1_ density in atrium, mean RMP was more negative in LV than in RA. However, the 2.5-fold higher I_K1_ related to only a small difference in mean RMP and, both in patch-clamp and sharp microelectrode measurements in intact tissue, individual RMP values largely overlapped between LV and RA. Thus, the power of RMP to discriminate atrial from ventricular CMs was modest. Tissues with an RMP negative to approximately −82 mV had an 80% probability to be correctly classified as ventricles. However, they represent less than 20% of ventricular preparations. Mean RMP values in EHT were similar to RA and LV. Together with the atrial-like I_K1_ densities, the atrial-like RMP would suggest an electrophysiological phenotype close to human RA. Yet, this interpretation is at odds with the lack of I_K,ACh_ in EHT from C25, and smaller I_K1_ density and more negative RMP in iCells, indicating that these cells exhibit a mixed phenotype. RMP in EHT was almost exactly between LV and RA, but not significantly different from either. Therefore, RMP may not be a useful parameter to decide if EHT may possess atrial or ventricular I_K1_. In addition, it should be emphasized that I_K1_ density alone does not determine RMP, but the relation of all conductances present, such as background sodium and calcium currents, NCX, and Na^+^/K^+^-ATPase (Maleckar et al., 2009), many of them not yet studied in detail in hiPSC-CMs.

It is common use in the stem cell field to classify hiPSC-CMs as atrial or ventricular like according to their AP duration (Doss et al., 2012, Liang et al., 2013, Ma et al., 2011, Ma et al., 2013, Moretti et al., 2010, Vaidyanathan et al., 2016). Such assumptions are based on data from different studies reporting APD_90_ values for human heart (Chandler et al., 2009, Dobrev et al., 2001, Drouin et al., 1998, Jakob et al., 1989, Li et al., 1998, Verkerk et al., 2007a, Verkerk et al., 2007b, Wang et al., 1993), which are difficult to compare for methodological reasons. Here, we present a large number of sharp microelectrode data, which were obtained under identical recording conditions. While mean APD_90_ values were indeed shorter in RA than in LV, individual values again largely overlapped, questioning whether measurements of APD_90_ are indeed helpful to discriminate between LV and RA in individual recordings.

Other approaches were used for a more precise discrimination between atrial- and ventricular-like APs such as APD_90_/APD_50_ (Chen et al., 2017, Dorn et al., 2015, Liang et al., 2013, Zhang et al., 2012). The early phase of repolarization in human heart is dominated by transient potassium outward currents. In contrast to ventricular CMs, the transient outward currents in atrial CMs exhibit a long-lasting component, which results in a larger repolarization fraction (Amos et al., 1996). We found a wide scattering of repolarization fraction in individual RA CMs. This finding is in line with the reported wide heterogeneity of transient outward currents in RA CMs (Amos et al., 1996). The repolarization fraction measured by sharp microelectrode in tissues likely integrates different AP shapes from individual cells and therefore the scatter is smaller. This interpretation is supported by the observation that mean values did not differ between individual isolated RA CMs and intact RA trabeculae. The same holds true for EHT. The smaller scatter in intact tissue allowed almost perfect discrimination between RA and LV and, according to this parameter, EHT resembled LV more than RA.

### Limitations

The present results have been obtained from two hiPSC lines (C25, iCell) and may differ in from other hiPSC lines. The points we want to make with this paper are (1) that patch clamping of hiPSC-CMs can underestimate the “true” RMP (as substantiated by sharp microelectrode data) and (2) that hiPSC-CMs can express relatively normal (human RA-like) I_K1_. The differences in I_K1_ density compared with earlier data are likely explained by donor-dependent differences between the hiPSC lines and/or by different culture conditions such as different media, culture time, or extracellular matrix as suggested previously (Doss et al., 2012, Herron et al., 2016). A more systematic comparison of different cell lines and different differentiation protocols and culture conditions is warranted. For technical reasons, I_K1_ was measured in isolated CMs, but RMP, APD_90_, and repolarization fraction in intact tissues/3D EHTs. Thus, we cannot exclude that the physiological RMP is a consequence of the EHT culture format and does not reflect that of ML-cultured hiPSC-CMs. Another question is how reliable patch-clamp protocols are and if they reflect physiology. Here we have used protocols applied previously for many years in human atrial CMs (Dobrev et al., 2001). The human CMs used in this study were isolated from heart tissues from patients undergoing open heart surgery for various cardiac diseases. While most RA samples were from patients with normal ventricular function, the LV samples necessarily were from patients with severe forms of heart failure. Thus, we cannot exclude that the measurements are influenced by the heart failure phenotype. Finally, RMP is not exclusively determined by I_K1_ conductance, because it was reported that over 70% block of I_K1_ (10 μM BaCl_2_) does not influence RMP in human LV tissue using sharp microelectrode technique (Jost et al., 2013). Therefore, we cannot rule out that culture-related changes in other background currents contribute to the physiological RMP measured in EHT.

### Conclusions

hiPSC-CMs from the C25 line possess robust I_K1_ currents, which, in ML, reached values of human LV CMs and, in EHT, that of RA CMs. I_K1_ density was 2-fold smaller in iCells than in C25, but was still associated with RA-like RMP in EHT. Technical issues related to patch clamping of small cells probably contribute to the reported low RMP in hiPSC-CMs. hiPSC-CMs exhibit features of both an atrial (I_K1_ and RMP) and ventricular phenotype (absence of I_K,ACh_ and low repolarization fraction). Low I_K1_ and depolarized RMP are not inherent characteristics of hiPSC-CMs.

## Experimental Procedures

### Differentiation of hiPSC-CM and EHT Generation

The undifferentiated hiPSC line C25 (a kind gift from Alessandra Moretti, Munich, Germany) was expanded in FTDA medium (Frank et al., 2012) and differentiated in a three-step protocol based on growth factors and a small-molecule Wnt inhibitor DS07 (a kind gift from Dennis Schade, Dortmund, Germany), as published previously (Lanier et al., 2012, Mannhardt et al., 2016). Details are given in the Supplemental Information.

### Dissociation of hiPSC-CMs from ML and EHT

After culturing hiPSC-CMs in ML and EHT for 28 days, cells were isolated with collagenase type II (200 U/mL, Worthington Biochemical, Lakewood, NJ, USA, LS004176 in HBSS minus Ca^2+/^Mg^2+^, Gibco 14175-053 and 50 μM CaCl_2_) for 3 hr (ML) and 5 hr (EHT). At least three different batches of hiPSC-CMs were used. Details are given in the Supplemental Information.

### Human Samples

Myocardial tissue was obtained from patients undergoing cardiac surgery at the University Heart Center Hamburg. The study followed the declaration of Helsinki. All patients gave written informed consent. Atrial and ventricular CMs were isolated and prepared as described previously (Dobrev et al., 2001) (Figures S4D and S4E). Details are given in the Supplemental Information.

### Voltage Clamp Recordings (K^+^ Currents)

Inwardly rectifying K^+^ currents were measured at room temperature, using the *whole-cell* configuration of the patch-clamp technique. The Axopatch 200B amplifier (Axon Instruments, Foster City, CA, USA) and ISO2 software were used for data acquisition and analysis (MFK, Niedernhausen, Germany). Details are given in the Supplemental Information.

### Current Clamp Recordings

Action potentials were recorded using the perforated patch (amphotericin B) configuration of the patch-clamp technique. The Axopatch 200B (Axon Instruments) was set to current-clamp mode and the experiments performed at 37°C, 1 Hz. Details are given in the Supplemental Information.

### Sharp Microelectrode Recordings

Sharp microelectrodes were used to record action potentials in RA and LV trabeculae and in intact EHT. The experiments were performed at 37°C. Details are given in the Supplemental Information.

### Drugs

All drugs and chemicals were obtained from Sigma-Aldrich (St. Louis, MO, USA).

## Author Contributions

A. H., A.U.U., I.M., M.D.L., A.L., C.N., and K.B. performed the research. A. H., A.U.U., A. H., A.L., N.J., T.E., and T.C. planned the experiments. A. H., M.D.L., I.M., K.B., C.N., A.L., and E.W. analyzed the results. A. H., T.E., and T.C. wrote the manuscript. All authors approved the final version of the manuscript.

## Supplemental Information

Document S1. Supplemental Experimental Procedures, Figures S1–S4, and Tables S1–S4

Document S2. Article plus Supplemental Information

## References

[bib1] Amos G.J., Wettwer E., Metzger F., Li Q., Himmel H.M., Ravens U. (1996). Differences between outward currents of human atrial and subepicardial ventricular myocytes. J. Physiol..

[bib2] Anumonwo J.M., Lopatin A.N. (2010). Cardiac strong inward rectifier potassium channels. J. Mol. Cell. Cardiol..

[bib3] Burridge P.W., Keller G., Gold J.D., Wu J.C. (2011). Review production of de novo cardiomyocytes: human pluripotent stem cell differentiation and direct reprogramming. Cell Stem Cell.

[bib4] Chandler N.J., Greener I.D., Tellez J.O., Inada S., Musa H., Molenaar P., DiFrancesco D., Baruscotti M., Longhi R., Anderson R.H. (2009). Molecular architecture of the human sinus node insights into the function of the cardiac pacemaker. Circulation.

[bib5] Chen Z., Xian W., Bellin M., Dorn T., Tian Q., Goedel A., Dreizehnter L., Schneider C.M., Ward-van Oostwaard D., Ng J.K.M. (2017). Subtype-specific promoter-driven action potential imaging for precise disease modelling and drug testing in hiPSC-derived cardiomyocytes. Eur. Heart J..

[bib6] Christoforou N., Liau B., Chakraborty S., Chellapan M., Bursac N., Leong K.W. (2013). Induced pluripotent stem cell-derived cardiac progenitors differentiate to cardiomyocytes and form biosynthetic tissues. PLoS One.

[bib7] Cordeiro J.M., Zeina T., Goodrow R., Kaplan A.D., Thomas L.M., Nesterenko V.V., Treat J.A., Hawel L., Byus C., Bett G.C. (2015). Regional variation of the inwardly rectifying potassium current in the canine heart and the contributions to differences in action potential repolarization. J. Mol. Cell. Cardiol..

[bib8] Davis R.P., Casini S., van den Berg C.W., Hoekstra M., Remme C.A., Dambrot C., Salvatori D., Oostwaard D.W., Wilde A.A., Bezzina C.R. (2012). Recapitulate electrophysiological characteristics of an overlap syndrome of cardiac sodium channel disease. Circulation.

[bib9] Dobrev D., Graf E., Wettwer E., Himmel H.M., Hála O., Doerfel C., Christ T., Schüler S., Ravens U. (2001). Molecular basis of downregulation of g-protein-coupled inward rectifying K+ current IK,ACh in chronic human atrial fibrillation. Circulation.

[bib10] Dobrzynski H., Marples D.D., Musa H., Yamanushi T.T., Henderson Z., Takagishi Y., Honjo H., Kodama I., Boyett M.R. (2001). Distribution of the muscarinic K+ channel proteins Kir3.1 and Kir3.4 in the ventricle, atrium, and sinoatrial node of heart. J. Histochem. Cytochem..

[bib11] Dorn T., Goedel A., Lam J.T., Haas J., Tian Q., Herrmann F., Bundschu K., Dobreva G., Schiemann M., Dirschinger R. (2015). Direct Nkx2-5 transcriptional repression of isl1 controls cardiomyocyte subtype identity. Stem Cells.

[bib12] Doss M.X., Di Diego J.M., Goodrow R.J., Wu Y., Cordeiro J.M., Nesterenko V.V., Barajas-Martínez H., Hu D., Urrutia J., Desai M. (2012). Maximum diastolic potential of human induced pluripotent stem cell-derived cardiomyocytes depends critically on IKr. PLoS One.

[bib13] Drouin E., Lande G., Charpentier F. (1998). Amiodarone reduces transmural heterogeneity of repolarization in the human heart. J. Am. Coll. Cardiol..

[bib14] Du D.T., Hellen N., Kane C., Terracciano C.M. (2015). Action potential morphology of human induced pluripotent stem cell-derived cardiomyocytes does not predict cardiac chamber specificity and is dependent on cell density. Biophys. J..

[bib15] Eder A., Hansen A., Uebeler J., Schulze T., Neuber C., Schaaf S., Yuan L., Christ T., Vos M.A., Eschenhagen T. (2014). Effects of proarrhythmic drugs on relaxation time and beating pattern in rat engineered heart tissue. Basic Res. Cardiol..

[bib16] Frank S., Zhang M., Schöler H.R., Greber B. (2012). Small molecule-assisted, line-independent maintenance of human pluripotent stem cells in defined conditions. PLoS One.

[bib17] Giles W.R., Noble D. (2016). Rigorous phenotyping of cardiac iPSC preparations requires knowledge of their resting potential(s). Biophys. J..

[bib18] Guo J., Mitsuiye T., Noma A. (1997). The sustained inward current in sino-atrial node cells of Guinea-pig heart. Pflugers Arch..

[bib19] Heidbüchel H., Vereecke J., Carmeliet E. (1987). The electrophysiological effects of acetylcholine in single human atrial cells. J. Mol. Cell. Cardiol..

[bib20] Herron T.J., Da Rocha A.M., Campbell K.F., Ponce-Balbuena D., Willis B.C., Guerrero-Serna G., Liu Q., Klos M., Musa H., Zarzoso M. (2016). Extracellular matrix-mediated maturation of human pluripotent stem cell-derived cardiac monolayer structure and electrophysiological function. Circ. Arrhythm. Electrophysiol..

[bib21] Hibino H., Inanobe A., Furutani K., Murakami S., Findlay I., Kurachi Y. (2010). Inwardly rectifying potassium channels: their structure, function, and physiological roles. Physiol. Rev..

[bib22] Itzhaki I., Maizels L., Huber I., Zwi-Dantsis L., Caspi O., Winterstern A., Feldman O., Gepstein A., Arbel G., Hammerman H. (2011). Modelling the long QT syndrome with induced pluripotent stem cells. Nature.

[bib23] Jakob H., Oelert H., Rupp J., Nawrath H. (1989). Functional role of cholinoceptors and purinoceptors in human isolated atrial and ventricular heart muscle. Br. J. Pharmacol..

[bib24] Jost N., Virág L., Comtois P., Ördög B., Szuts V., Seprényi G., Bitay M., Kohajda Z., Koncz I., Nagy N. (2013). Ionic mechanisms limiting cardiac repolarization reserve in humans compared to dogs. J. Physiol..

[bib25] Karakikes I., Stillitano F., Nonnenmacher M., Tzimas C., Sanoudou D., Termglinchan V., Kong C.W., Rushing S., Hansen J., Ceholski D. (2015). Correction of human phospholamban R14del mutation associated with cardiomyopathy using targeted nucleases and combination therapy. Nat. Commun..

[bib26] Keizer J., Magnus G. (1989). ATP-sensitive potassium channel and bursting in the pancreatic beta cell. Biophys. J..

[bib27] Kijlstra J.D., Hu D., Mittal N., Kausel E., Van Der Meer P., Garakani A., Domian I.J. (2015). Integrated analysis of contractile kinetics, force generation, and electrical activity in single human stem cell-derived cardiomyocytes. Stem Cell Reports.

[bib28] Koumi S., Backer C.L., Arentzen C.E. (1995). Characterization of inwardly rectifying K+ channel in human cardiac myocytes. Circulation.

[bib29] Krapivinsky G., Gordon E.A., Wickman K., Velimirović B., Krapivinsky L., Clapham D.E. (1995). The G-protein-gated atrial K+ channel IKACh is a heteromultimer of two inwardly rectifying K(+)-channel proteins. Nature.

[bib30] Lanier M., Schade D., Willems E., Tsuda M., Spiering S., Kalisiak J., Mercola M., Cashman J.R. (2012). Wnt inhibition correlates with human embryonic stem cell cardiomyogenesis: a structure-activity relationship study based on inhibitors for the Wnt response. J. Med. Chem..

[bib31] Lemoine M.D., Mannhardt I., Breckwoldt K., Prondzynski M., Flenner F., Ulmer B., Hirt M.N., Neuber C., Horváth A., Kloth B. (2017). Human iPSC-derived cardiomyocytes cultured in 3D engineered heart tissue show physiological upstroke velocity and sodium current density. Sci. Rep..

[bib32] Levy M.N. (1971). Sympathetic-parasympathetic interactions in the heart. Circ. Res..

[bib33] Li G.R., Feng J., Yue L., Carrier M. (1998). Transmural heterogeneity of action potentials and Ito1 in myocytes isolated from the human right ventricle. Am. J. Physiol..

[bib34] Liang P., Lan F., Lee A.S., Gong T., Sanchez-Freire V., Wang Y., Diecke S., Sallam K., Knowles J.W., Wang P.J. (2013). Drug screening using a library of human induced pluripotent stem cell-derived cardiomyocytes reveals disease-specific patterns of cardiotoxicity. Circulation.

[bib35] Liang P., Sallam K., Wu H., Li Y., Itzhaki I., Garg P., Zhang Y., Termglichan V., Lan F., Gu M. (2016). Patient-specific and genome-edited induced pluripotent stem cell-derived cardiomyocytes elucidate single-cell phenotype of brugada syndrome. J. Am. Coll. Cardiol..

[bib36] Liu G.X., Derst C., Schlichthörl G., Heinen S., Seebohm G., Brüggemann A., Kummer W., Veh R.W., Daut J., Preisig-Müller R. (2001). Comparison of cloned Kir2 channels with native inward rectifier K+ channels from Guinea-pig cardiomyocytes. J. Physiol..

[bib37] Lu H.R., Whittaker R., Price J.H., Vega R., Pfeiffer E.R., Cerignoli F., Towart R., Gallacher D.J. (2015). High throughput measurement of Ca++ dynamics in human stem cell-derived cardiomyocytes by kinetic image cytometery: a cardiac risk assessment characterization using a large panel of cardioactive and inactive compounds. Toxicol. Sci..

[bib38] Ma J., Guo L., Fiene S.J., Anson B.D., Thomson J.A., Kamp T.J., Kolaja K.L., Swanson B.J., January C.T., Kl K. (2011). High purity human-induced pluripotent stem cell-derived cardiomyocytes: electrophysiological properties of action potentials and ionic currents. Am. J. Physiol. Heart Circ. Physiol..

[bib39] Ma D., Wei H., Zhao Y., Lu J., Li G., Sahib N.B., Tan T.H., Wong K.Y., Shim W., Wong P. (2013). Modeling type 3 long QT syndrome with cardiomyocytes derived from patient-specific induced pluripotent stem cells. Int. J. Cardiol..

[bib40] Maddah M., Heidmann J.D., Mandegar M.A., Walker C.D., Bolouki S., Conklin B.R., Loewke K.E. (2015). A non-invasive platform for functional characterization of stem-cell-derived cardiomyocytes with applications in cardiotoxicity testing. Stem Cell Reports.

[bib41] Major P., Baczkó I., Hiripi L., Odening K.E., Juhász V., Kohajda Z., Horváth A., Seprényi G., Kovács M., Virág L. (2016). A novel transgenic rabbit model with reduced repolarization reserve: long QT syndrome caused by a dominant-negative mutation of the KCNE1 gene. Br. J. Pharmacol..

[bib42] Maleckar M.M., Greenstein J.L., Giles W.R., Trayanova N.A. (2009). K+ current changes account for the rate dependence of the action potential in the human atrial myocyte. Am. J. Physiol. Heart Circ. Physiol..

[bib43] Mannhardt I., Breckwoldt K., Letuffe-Brenière D., Schaaf S., Schulz H., Neuber C., Benzin A., Werner T., Eder A., Schulze T. (2016). Human engineered heart tissue: analysis of contractile force. Stem Cell Reports.

[bib44] Moretti A., Bellin M., Welling A., Jung C.B., Lam J.T., Bott-Flügel L., Dorn T., Goedel A., Höhnke C., Hofmann F. (2010). Patient-specific induced pluripotent stem-cell models for long-QT syndrome. N. Engl. J. Med..

[bib45] Meijer van Putten R.M., Mengarelli I., Guan K., Zegers J.G., Van Ginneken A.C., Verkerk A.O., Wilders R. (2015). Ion channelopathies in human induced pluripotent stem cell derived cardiomyocytes: a dynamic clamp study with virtual IK1. Front. Physiol..

[bib46] Rizzetto R., Rocchetti M., Sala L., Ronchi C., Villa A., Ferrandi M., Molinari I., Bertuzzi F., Zaza A. (2015). Late sodium current (INaL) in pancreatic β-cells. Pflugers Arch..

[bib47] Rorsman Patrik T.G. (1986). Calcium and delayed potassium currents in mouse pancreatic beta-cells under voltage-clamp conditions. J. Physiol..

[bib48] Sakmann B., Noma A., Trautwein W. (1983). Acetylcholine activation of single muscarinic K+ channels in isolated pacemaker cells of the mammalian heart. Nature.

[bib49] Schneider M.F., Chandler W.K. (1976). Effects of membrane potential on the capacitance of skeletal muscle fibers. J. Gen. Physiol..

[bib50] Schram G., Pourrier M., Melnyk P., Nattel S. (2002). Differential distribution of cardiac ion channel expression as a basis for regional specialization in electrical function. Circ. Res..

[bib51] Schram G., Pourrier M., Wang Z., White M., Nattel S. (2003). Barium block of Kir2 and human cardiac inward rectifier currents: evidence for subunit-heteromeric contribution to native currents. Cardiovasc. Res..

[bib52] Szentandrássy N., Kistamás K., Hegyi B., Horváth B., Ruzsnavszky F., Váczi K., Magyar J., Bányász T., Varró A., Nánási P.P. (2015). Contribution of ion currents to beat-to-beat variability of action potential duration in canine ventricular myocytes. Pflugers Arch..

[bib53] Tamargo J., Caballero R., Gómez R., Valenzuela C., Delpón E. (2004). Pharmacology of cardiac potassium channels. Cardiovasc. Res..

[bib54] Uzun A.U., Mannhardt I., Breckwoldt K., Horváth A., Johannsen S.S., Hansen A., Eschenhagen T., Christ T. (2016). Ca2+-currents in human induced pluripotent stem cell-derived cardiomyocytes effects of two different culture conditions. Front. Pharmacol..

[bib55] Vaidyanathan R., Markandeya Y.S., Kamp T.J., Makielski J.C., January C.T., Eckhardt L.L. (2016). IK1-enhanced human-induced pluripotent stem cell-derived cardiomyocytes: an improved cardiomyocyte model to investigate inherited arrhythmia syndromes. Am. J. Physiol. Heart Circ. Physiol..

[bib56] Varró A., Nánási P.P., Lathrop D.A. (1993). Potassium currents in isolated human atrial and ventricular cardiocytes. Acta Physiol. Scand..

[bib57] Verkerk A.O., van Borren M.M., Peters R.J., Broekhuis E., Lam K.Y., Coronel R., De Bakker J.M., Tan H.L., Wilders R. (2007). Single cells isolated from human sinoatrial node: action potentials and numerical reconstruction of pacemaker current. Conf. Proc. IEEE Eng. Med. Biol. Soc..

[bib58] Verkerk A.O., Wilders R., van Borren M.M., Peters R.J., Broekhuis E., Lam K., Coronel R., de Bakker J.M., Tan H.L. (2007). Pacemaker current (If) in the human sinoatrial node. Eur. Heart J..

[bib59] Wang Z., Fermini B., Nattel S. (1993). Delayed rectifier outward current and repolarization in human atrial myocytes. Circ. Res..

[bib60] Wang Z., Yue L., White M., Pelletier G., Nattel S. (1998). Differential distribution of inward rectifier potassium channel transcripts in human atrium versus ventricle. Circulation.

[bib61] Weinberger F., Breckwoldt K., Pecha S., Kelly A., Geertz B., Starbatty J., Yorgan T., Cheng K.H., Lessmann K., Stolen T. (2016). Cardiac repair in Guinea pigs with human engineered heart tissue from induced pluripotent stem cells. Sci. Transl. Med..

[bib62] Wilson J.R., Clark R.B., Banderali U., Giles W.R. (2011). Measurement of the membrane potential in small cells using patch clamp methods. Channels (Austin).

[bib63] Yamada M., Inanobe A., Kurachi Y. (1998). G protein regulation of potassium ion channels. Pharmacol. Rev..

[bib64] Yazawa M., Hsueh B., Jia X., Pasca A.M., Bernstein J.A., Hallmayer J., Dolmetsch R.E. (2011). Using induced pluripotent stem cells to investigate cardiac phenotypes in Timothy syndrome. Nature.

[bib65] Zhang H., Zou B., Yu H., Moretti a., Wang X., Yan W., Babcock J.J., Bellin M., McManus O.B., Tomaselli G. (2012). Modulation of hERG potassium channel gating normalizes action potential duration prolonged by dysfunctional KCNQ1 potassium channel. Proc. Natl. Acad. Sci. USA.

[bib66] Zhang J., Wilson G.F., Soerens A.G., Koonce C.H., Yu J., Palecek S.P., Thomson J.A., Kamp T.J. (2009). Functional cardiomyocytes derived from human induced pluripotent stem cells. Circ. Res..

[bib67] Zhang M., D'Aniello C., Verkerk A.O., Wrobel E., Frank S., Ward-van Oostwaard D., Piccini I., Freund C., Rao J., Seebohm G. (2014). Recessive cardiac phenotypes in induced pluripotent stem cell models of Jervell and Lange-Nielsen syndrome: disease mechanisms and pharmacological rescue. Proc. Natl. Acad. Sci. USA.

