# Supplementary material for: Low Resting Membrane Potential and Low Inward Rectifier Potassium Currents Are Not Inherent Features of hiPSC-Derived Cardiomyocytes
Source: Stem Cell Reports. 2018 Feb 8;10(3):822–33. doi: 10.1016/j.stemcr.2018.01.012 (PMC5918194; doi:10.1016/j.stemcr.2018.01.012)
Supplement: Document S1. Supplemental Experimental Procedures, Figures S1–S4, and Tables S1–S4 [file mmc1.pdf]

Stem Cell Reports, Volume *10*

Supplemental Information

Low Resting Membrane Potential and Low Inward Rectifier Potassium Currents Are Not Inherent Features of hiPSC-Derived Cardiomyocytes

András Horváth, Marc D. Lemoine, Alexandra Löser, Ingra Mannhardt, Frederik Flenner, Ahmet Umur Uzun, Christiane Neuber, Kaja Breckwoldt, Arne Hansen, Evaldas Girdauskas, Hermann Reichenspurner, Stephan Willems, Norbert Jost, Erich Wettwer, Thomas Eschenhagen, and Torsten Christ

Supplemental information

Supplemental Data

Figure S1. Related to Figure 1, 2 and 3

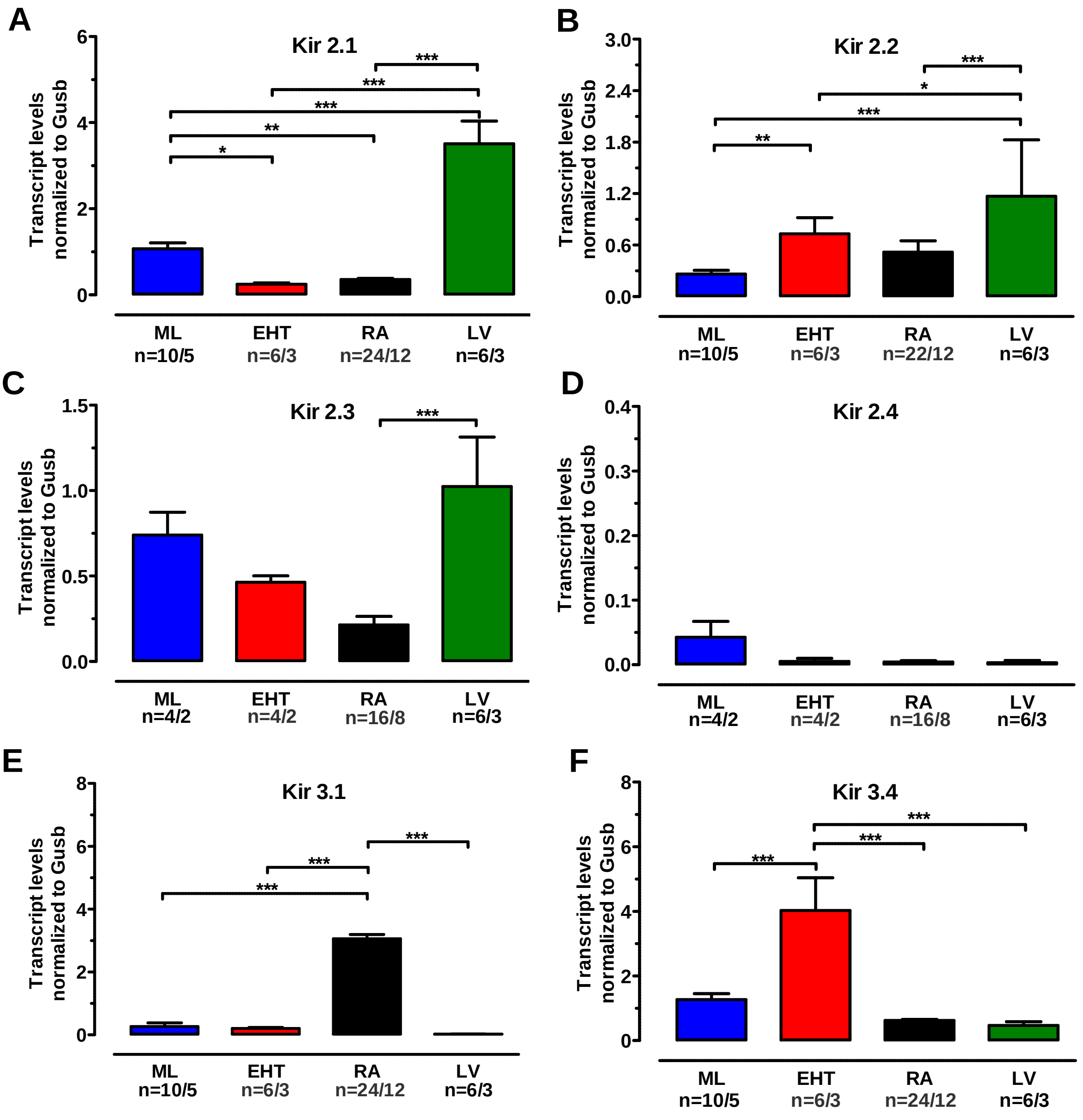

Figure S1. Expression of Kir channels in hiPSC-CM and human adult CM (Related to Figure 1, 2 and 3).

Transcript levels of I_{K1} channel-forming subunits Kir 2.1, 2.2, 2.3 and Kir2.4 and transcript levels of $I_{K.ACh}$ channel-forming subunits Kir 3.1 and Kir 3.4 were normalized to housekeeping gene (GUSB). *P<0.05, **P<0.01, ***P<0.001 (One-way Anova followed by Bonferroni test). n/n=number of experiment/number of isolations in hiPSC-CM and number of experiment/number of patients in RA and LV.

Figure S2. Related to Figure 5.

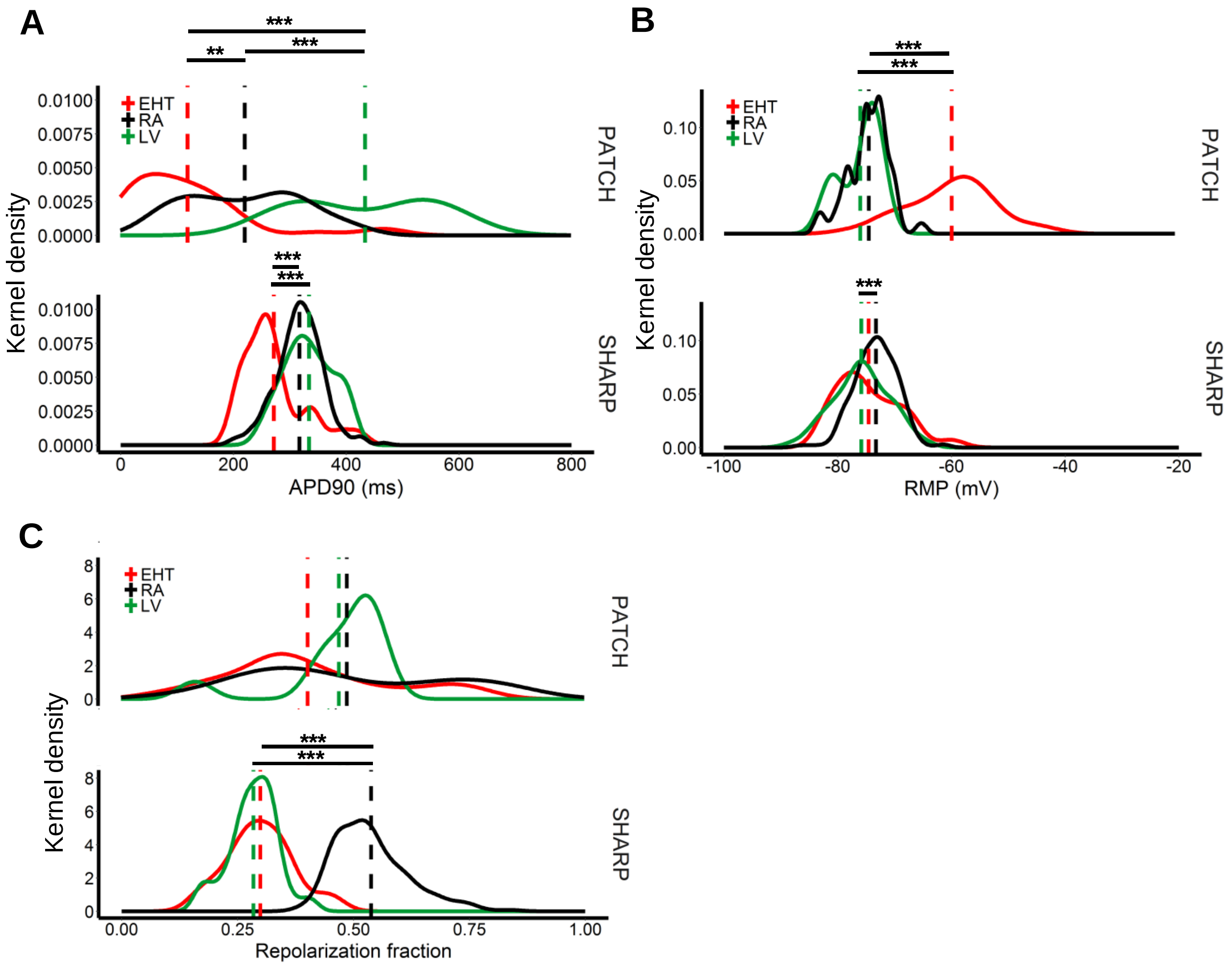

Figure S2. Frequency distribution of APD_{90}, RMP and repolarization fraction in EHT, RA and LV.

Frequency distribution expressed as kernel density and mean values for APD_{90} (A) RMP (B) and repolarization fraction (C). Data obtained by patch clamping of isolated CM from EHT, RA and LV are given in the upper panels, measured by sharp microelectrodes in intact tissues from EHT, RA and LV are given in the lower panels. *P<0.05, **P<0.01, ***P<0.001 (One-way ANOVA followed by Kruskal-Wallis test).

Figure S3.

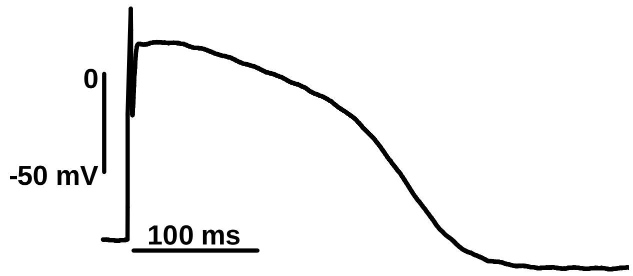

Figure S3. Original recording from a single hiPSC-CM using sharp microelectrode.

Figure S4.

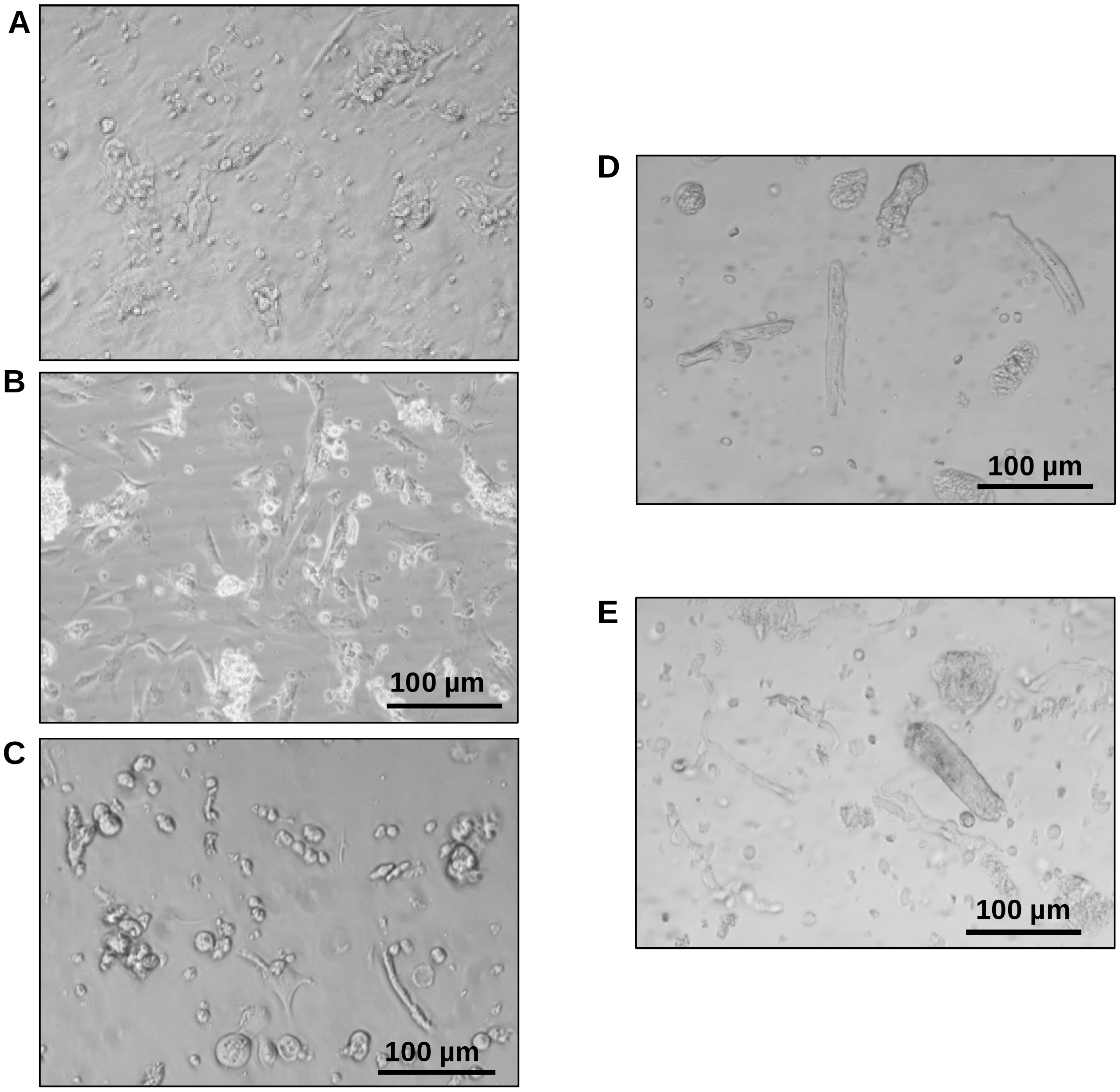

Figure S4. Images of isolated hiPSC-CM and adult CM.

A: C25-ML, B: C25-EHT, C: CDI-EHT, D: RA, E: LV

2. Supplemental Tables

Table S1. I_{K1} densities in pA/pF, descriptive values.

	**n**	**mean**	**SD**	**median**	**min**	**max**	**skew**	**kurtosis**
**ML**	71	32.7	36.2	19.70	0.4	182	2.12	4.824
**EHT**	60	14.1	12.0	8.98	0.0	50	1.20	0.418
**RA**	55	14.0	11.0	11.85	0.6	55	1.17	1.916
**LV**	14	40.5	28.2	33.52	13.5	118	1.40	1.413

ML: Monolayer, EHT: Engineered Heart Tissue, RA: Right Atrium, LV: Left Ventricle

Table S2. APD_{90} in ms, descriptive values.

	**n**	**mean**	**SD**	**median**	**min**	**max**	**skew**	**kurtosis**
**EHT_Patch**	41	119.06	108.04	100.0	12.5	475.0	1.72	3.05
**RA_Patch**	41	220.68	102.68	220.0	60.0	410.0	0.07	–1.22
**LV_Patch**	10	434.00	122.04	440.0	270.0	600.0	–0.02	–1.89
**EHT_Sharp**	24	271.45	56.07	259.3	205.9	421.7	0.53	1.22
**RA_Sharp**	220	316.91	41.98	319.0	193.0	467.0	0.01	0.84
**LV_Sharp**	57	333.77	43.66	331.5	250.0	411.2	0.02	–0.99

EHT-Patch: Cardiomyocytes from Engineered Heart Tissue measured with patch clamp technique, RA-Patch: Cardiomyocytes from right atrium measured with patch clamp technique, LV-Patch: Cardiomyocytes from left ventricle measured with patch clamp technique, EHT-Sharp: Engineered Heart Tissue measured with sharp microelectrode technique, RA-Sharp: right atrium measured with sharp microelectrode technique, LV-Sharp: left ventricle measured with sharp microelectrode technique.

Table S3. RMP in mV, descriptive values.

	**n**	**mean**	**SD**	**median**	**min**	**max**	**skew**	**kurtosis**
**EHT_Patch**	41	–59.66	7.77	–58.00	–78.00	–42.00	–0.13	–0.25
**RA_Patch**	41	–74.37	3.65	–75.00	–83.00	–65.00	–0.30	0.31
**LV_Patch**	10	–75.87	3.54	–74.58	–81.78	–72.27	–0.62	–1.48
**EHT_Sharp**	24	–74.58	5.69	–76.75	–82.20	–60.00	0.60	0.18
**RA_Sharp**	220	–73.31	3.78	–73.00	–87.00	–61.00	–0.16	0.74

**LV_Sharp**	57	–75.87	4.96	–76.00	–88.00	–65.00	–0.09	–0.42

Same abbreviations are used as in Table S2.

Table S4. Repolarization fraction descriptive values.

	**n**	**mean**	**SD**	**median**	**min**	**max**	**skew**	**kurtosis**
**EHT_Patch**	41	0.40	0.18	0.36	0.07	0.77	0.46	–0.56
**RA_Patch**	41	0.49	0.21	0.43	0.09	0.86	0.26	–1.23
**LV_Patch**	10	0.47	0.12	0.52	0.16	0.55	–1.74	1.93
**EHT_Sharp**	24	0.32	0.07	0.29	0.17	0.45	0.71	0.15
**RA_Sharp**	220	0.54	0.08	0.52	0.38	0.84	0.92	0.82
**LV_Sharp**	57	0.28	0.05	0.29	0.17	0.41	–0.21	0.12

Same abbreviations are used as in Table S2.

3. Supplemental Experimental Procedures

3.1. Differentiation of hiPSC-CM and EHT generation

In brief, confluent undifferentiated cells were dissociated (0.5 mM EDTA; 10 min) and cultivated in spinner flasks (30*106 cells/100 ml; 40 rpm) for embryoid body formation overnight (Zweigerdt et al., 2011). Mesodermal differentiation was initiated in embryoid bodies over three days in suspension culture with growth factors (BMP-4 [R&D Systems, 314-BP], activin-A [R&D Systems, 338-AC], FGF2 [PeproTech, 100-18B]). Cardiac differentiation was performed either in adhesion or in suspension culture with Wnt-inhibitor DS07 (Lanier et al., 2012). Cells were cultured in a humidified temperature and gas-controlled incubator (37 °C, 5% CO_2, 5% O_2; 21% O_2 for final cardiac differentiation). At day 14 the spontaneously beating hiPSC-CM were dissociated with collagenase II (Worthington, LS004176; 200 U/ml, 3.5 h) and either cultured in ML or EHT format. For three-dimensional culture EHT were generated as previously described with 1x106 cells / 100 µl EHT (Schaaf et al., 2014). EHT as well as ML were cultured in a 37 °C, 7% CO_2, 40% O_2 humidified cell culture incubator with a medium consisting of DMEM (Biochrom; F0415), 10% heat-inactivated horse serum (Gibco 26050), 1% penicillin/streptomycin (Gibco 15140), insulin (10 µg/ml; Sigma I9278) and aprotinin (33 µg/ml; Sigma A1153). For further comparability, experiments were performed in parallel from the same batch of cells.

3.2 Quantification of transcript levels

The RNeasy® Plus Mini Kit (Qiagen, Venlo, The Netherlands) was used to isolate total RNA from human heart tissue, cardiomyocytes cultured in a ML and dissociated EHT. For quantification of transcript levels, cDNA was generated using the High-Capacity cDNA Reverse Transcription Kit (Applied Biosystems). Quantitative PCR was performed with the 5x HOT FIREPol® EvaGreen® qPCR Mix Plus (ROX) (Solis BioDyne, Tartu, Estonia) on ABI PRISM 7900HT Sequence detection system (Applied Biosystems, Foster City, California, USA). Relative transcript levels were calculated using the ΔCT-method with glucuronidase beta (Gusb) as a housekeeping gene.

3.3 Dissociation of hiPSC-CM from ML and EHT

Cells were plated on gelatine-coated (0.1%) glass coverslips (12 mm diameter, Carl Roth GmbH+Co, Karlsruhe, Germany) for 24–48 h before patch clamp experiments were performed (Figure S4A, B, C). To avoid changes in morphology and ion currents due to culture conditions as much as possible, the cells were investigated within 48 hours.

3.4 Voltage clamp recordings (K^+ currents)

Heat-polished pipettes were pulled from borosilicate filamented glass (Hilgenberg, Malsfeld, Germany). Tip resistances were 2.5–5 MΩ, seal resistances were 2–4 GΩ. The cells were investigated in a small perfusion chamber placed on the stage of an inverse microscope. To reduce the liquid junction potential an agarose bridge was placed into a solution containing high KCL. The experiments were performed with the following bath solution (in mM): NaCl 120, KCl 20, HEPES 10, $CaCl_2$ 2, $MgCl_2$ 1 and glucose 10 (pH 7.4, adjusted with NaOH)(Dobrev et al., 2005). Contaminating Ca^{2+} currents were suppressed with the selective L-type calcium channel blocker nifedipine (10 µM). For I_{K1} and $I_{K,ACh}$ the internal solution included (in mM): DL-Aspartate potassium salt 80, KCl 40, NaCl 8, HEPES 10, Mg-ATP 5, Tris-GTP 0.1, EGTA 5 and $CaCl_2$ 2, pH 7.4, adjusted with KOH (Dobrev et al., 2005). Inward Current amplitudes were determined as currents at –100 mV. A single concentration (2 µM) of the muscarinic receptor agonist carbachol (CCh) was used to evoke $I_{K,ACh}$.

3.5 Current clamp recordings

The Axopatch 200B (Axon Instruments, Foster City, CA, USA) was set to current clamp mode and the experiments performed at 37 °C, 1 Hz, with the following bath solution (in mM): NaCl 120, KCl 5.4, HEPES 10, $CaCl_2$ 2, $MgCl_2$ 1 and glucose 10 (pH 7.4, adjusted with NaOH). The internal solution was (in mM): DL-aspartate potassium salt 80, KCl 40, NaCl 8, HEPES 10, Mg-ATP 5, Tris-GTP 0.1, EGTA 5 and $CaCl_2$ 2 (pH 7.4, adjusted with KOH, Dobrev et al., 2001). The liquid junction potential was calculated by using the JPCalcW software (SDR Scientific, Sydney, Australia) to –12 mV, for both voltage and current clamp recordings. The tips the of pipettes were filled with the same solution, but supplemented with 20 µg/ml Amphotericin B to allow formation of perorated patch configuration within a few minutes. In action potential recordings the repolarization fraction was calculated by the following way: $(APD_{50}–APD_{90})/APD_{90}$(Du et al., 2015).

3.6 Sharp microelectrode recordings

Microelectrode tip resistances were 20 to 50 MΩ when filled with 3 mM KCl). Action potentials were elicited by field stimulation at 1 Hz: 0.5 ms stimulus, 50% above threshold intensity. The following bath solution was used (in mM): NaCl 125, KCl 5.4, $MgCl_2$ 0.6, $CaCl_2$ 1, NaH_2PO_4 0.4, NaH_2CO_3 22, and glucose 5.5 and was equilibrated with O2–CO2 (95:5).

3.7 Sharp microelectrode recordings in single hiPSC-CM

Sharp microelectrodes were used to record action potentials in isolated single hiPSC-CM. Microelectrode tip resistances were 20 to 50 MΩ when filled with 3 mM KCl). Action potentials were elicited by field stimulation at 1 Hz: 0.5 ms stimulus. The following bath solution was used (in mM): NaCl 120, KCl 5.4, HEPES 10, $CaCl_2$ 2, $MgCl_2$ 1 and glucose 10 (pH 7.4, adjusted with NaOH). The experiments were performed at 37 °C.

3.8 Statistics

Results are presented as mean values±SEM. Curve fitting was performed by using the GraphPad Prism Software 5.02 (GraphPad Software, San Diego, CA, USA). Statistical differences were evaluated by using the Student's t-test (paired or unpaired) or repeated measures ANOVA, followed by Bonferroni test, where appropriate. A value of $p<0.05$ was considered to be statistically significant. Linear correlations, concentration-response curves were fitted to mean data points and –logIC50 values were compared by F-test using the GraphPad Prism Software 5.02. Analyses of frequency distribution were performed using R (ver. 3.3.1) (R Core Team, 2013). Please note that the statistical term "Kernel density estimation" is used in Fig. S2, with, a "kernel" defined as a probability density function which must possess the following properties: even non-negative, real-valued and its definite integral over its support set must equal to 1. Therefore, "kernel density estimation" is a non-parametric method, which allows estimating the probability density function of a random variable (http://scikit-learn.org/ stable/modules/density.html). The kurtosis describes the tail shape of the data distribution (http://www.r-tutor.com/elementary-statistics/numerical-measures/kurtosis). The skewness is a measure of symmetry (http://www.r-tutor.com/elementary-statistics/numerical-measures/skewness).

4. Supplemental References

Dobrev, D., Friedrich, A., Voigt, N., Jost, N., Wettwer, E., Christ, T., Knaut, M., and Ravens, U. (2005). The G protein-gated potassium current IK,ACh is constitutively active in patients with chronic atrial fibrillation. Circulation

112, 3697–3706.

Du, D.T.M., Hellen, N., Kane, C., and Terracciano, C.M.N. (2015). Action potential morphology of human induced pluripotent stem cell-derived cardiomyocytes does not predict cardiac chamber specificity and is dependent on cell density. Biophys. J. *108*, 1–4.

Lanier, M., Schade, D., Willems, E., Tsuda, M., Spiering, S., Kalisiak, J., Mercola, M., and Cashman, J.R. (2012). Wnt Inhibition Correlates with Human Embryonic Stem Cell Cardiomyogenesis: A Structure − Activity Relationship Study Based on Inhibitors for the Wnt Response. J. Med. Chem. *55*, 697–708.

Schaaf, S., Eder, A., Vollert, I., Stöhr, A., Hansen, A., and Eschenhagen, T. (2014). Generation of Strip-Format Fibrin-Based Engineered Heart Tissue (EHT). In Cardiac Tissue Engineering: Methods and Protocols, M. Radisic, and D.L. Black III, eds. (New York, NY: Springer New York), pp. 121–129.

Zweigerdt, R., Olmer, R., Singh, H., Haverich, A., and Martin, U. (2011). Scalable expansion of human pluripotent stem cells in suspension culture. Nat. Protoc. *6*, 689–700.
